# Facile Fabrication of Three-Dimensional Hydrogel Film with Complex Tissue Morphology

**DOI:** 10.3390/bioengineering8110164

**Published:** 2021-10-27

**Authors:** Young-Hyeon An, Su-Hwan Kim

**Affiliations:** 1 BioMax/N-Bio Institute, Seoul National University, Seoul 08826, Korea; ayh015@snu.ac.kr; 2 Department of Chemical Engineering (BK 21 FOUR), Dong-A University, Busan 49315, Korea

**Keywords:** tissue structure molding, catechol-modified alginate, implantable scaffold, local delivery, cardiac tissue engineering

## Abstract

In this study, we proposed a simple and easy method for fabricating a three-dimensional (3D) structure that can recapitulate the morphology of a tissue surface and deliver biological molecules into complex-shaped target tissues. To fabricate the 3D hydrogel film structure, we utilized a direct tissue casting method that can recapitulate tissue structure in micro-/macroscale using polydimethylsiloxane (PDMS). A replica 3D negative mold was manufactured by a polyurethane acrylate (PUA)-based master mold. Then, we poured the catechol-conjugated alginate (ALG-C) solution into the mold and evaporated it to form a dried film, followed by crosslinking the film using calcium chloride. The ALG-C hydrogel film had a tensile modulus of 725.2 ± 123.4 kPa and maintained over 95% of initial weight after 1 week without significant degradation. The ALG-C film captured over 4.5 times as much macromolecule (FITC-dextran) compared to alginate film (ALG). The cardiomyoblast cells exhibited high cell viability over 95% on ALG-C film. Moreover, the ALG-C film had about 70% of surface-bound lentivirus (1% in ALG film), which finally exhibited much higher viral transfection efficiency of GFP protein to C2C12 cells on the film than ALG film. In conclusion, we demonstrated a 3D film structure of biofunctionalized hydrogel for substrate-mediated drug delivery, and this approach could be utilized to recapitulate the complex-shaped tissues.

## 1. Introduction

Substrate-mediated drug delivery has been a promising approach for treating a wide spectrum of human diseases [1,2]. Recent advances in surface engineering have enabled the chemical modification of polymeric substrate to deliver biological supramolecules such as protein or viral particles into a local area [3,4]. In the field of cardiac tissue engineering, substrate-mediated approaches of biocompatible polymers have been widely applicable for ease of drug loading and delivery [5,6]. However, the big challenges in designing the substrate for in vivo delivery of drugs have been addressed in terms of the heterogeneous tissue surface, such as cardiac structure. Generally, cardiac tissue has a complex 3D structure where each extracellular matrix (ECM) layer has a different helical pitch [7,8]. The cardiomyocytes are circumferentially aligned, and all the surface of the cardiac tissue is curved in a macroscopic view [9,10]. So far, conventional substrates such as 2D flat cell sheet or nanofiber scaffold have inappropriate structures to adhere stably on the rough surface of cardiac tissue [11]. In addition, cardiac tissue has the capability of excitation that can cause rhythmical discharge and contraction [8], leading to the stimulation of cardiac muscle following active sinus beat [8]. Even though a two-dimensional scaffold can be stretchable or flexible, it might be readily dislocated or detached from the cardiac surface owing to the active contraction of cardiomyocytes.

Several studies have demonstrated artificial whole heart chambers by seeding cardiomyocytes with hydrogel in a ventricle-like shape or by using 3D printing technology [12,13,14]. MacQueen et al. have designed the whole heart chamber using rotary-jet spinning [13]. Nanofibrous ventricle structure has a similar micro-scale substructure to native cardiac tissue. Lee et al. have developed engineered heart tissue (EHT) casting ball-shaped polypropylene tubes in agarose hydrogel [12]. The EHT was filled with collagen, Matrigel, and cardiomyocytes, resulting in a pouch-like shape. Recent advances in 3D printing have also been applied in producing cardiac tissue [14]. Kupfer et al. have generated an artificial cardiac chamber using photo-crosslinkable hydrogel through 3D bioprinting [14]. Despite recent advances in this field, no approaches have yet been demonstrated to obtain a system that fully recapitulates the complexity of the cardiac tissue. In addition, the suggested approaches are time-consuming and need a variety of equipment. Moreover, such approaches aim to fabricate in situ cardiac organoids to replace animal models in clinical settings, which are not suitable for implantation for cardiac tissue regeneration.

Recent advances in mussel-inspired chemistry enable a high degree of tissue adhesiveness and protein adhesion without any catalyst [15,16,17,18]. The underlying mechanism of mussel chemistry in tissue adhesiveness has not been well determined, but the oxidative reaction of catechol moiety can be turned into reactive quinone adduct following interacting with any substrate [19]. The catechol group can interact with biological surfaces and proteins via non-covalent interaction [20]. When it comes to drug delivery application, catechol-conjugated hydrogels (e.g., alginate, hyaluronic acid, chitosan, etc.) have been used as drug carriers to deliver biological macromolecules [21,22,23,24]. The catechol group itself has shown little cytotoxicity or side effects on the native tissues. In addition, the scaffold for tissue engineering should support mechanical properties as well as biological features which can promote cellular behavior and flexibility. The presentation of the catechol group on hydrogel can enhance cellular attachment and ECM deposition. The scaffold with appropriate chemical and physical cues does support a cell-friendly environment and application for drug delivery. So far, a 3D film structure with catechol-conjugated hydrogel would be a promising system to deliver biological macromolecules into human tissue along with providing cell-friendly environments.

We proposed that a direct tissue casting method enables the production of a 3D tissue structure easily and efficiently without complex processes, such as soft lithography and 3D bioprinting. This study aims to develop an implantable substrate-mediated drug delivery platform that has complex surface morphology. To reconstruct the cardiac tissue with hydrogel, we first fabricated a cardiac-shaped PDMS mold using the direct tissue casting method. Then, the catechol-functionalized alginate hydrogel (ALG-C) was poured into the PDMS mold and dehydrated overnight. The final product was cardiac-tissue-shaped ALG-C film in 3D. The 3D structure has an optically transparent and highly tissue-mimetic structure without any flaw on the surface. Cardiomyoblast H9c2 cell line, which can be differentiated into functional cardiomyocytes, has been proved in several studies as a candidate cell source for cardiac tissue engineering [25,26,27]. As the permanent cardiac cell line, H9c2 cells have been fully characterized with biochemical and electrophysiological properties; thus, it was utilized as a model cell in this work. The primary interest of this study is to develop a 3D tissue-shaped scaffold for topically delivering biological drugs. The physicochemical properties of ALG-C film have revealed that it has suitable stiffness without any cytotoxicity of H9c2 cells. Moreover, model drugs were stably attached to the surface of the 3D film structure due to the catechol group. This study highlights that this direct tissue casting would be an alternative way for the multi-scale tissue mimetic strategy.

## 2. Materials and Methods

### 2.1. Fabrication of Cardiac Tissue-Shaped 3D Replica Structure

To fabricate the tissue structure replica, we introduced the tissue casting technique. At first, we collected cardiac tissues from mice (4 weeks old balb/c; OrientBio). All animal experiments were performed using protocols approved by the Seoul National University Institutional Animal Care and Use Committees (SNU-141229-3-9). After removing surface moisture using Kimwipes, cardiac tissue was put into polydimethylsiloxane (PDMS, Sylgard^TM^ 184, Dow Corning Corporation) precursor. Then, PDMS was cured for 4 h at 80 °C. The PDMS-based negative mold of the cardiac structure was produced after removing cardiac tissue. Next, polyurethane acrylate (PUA) was filled into a negative mold of cardiac tissue, followed by curing for 5 min using UV light. The PUA-based positive replica of cardiac tissue was used as a master mold. PDMS (Sylgard^TM^ 184, Dow Corning Corporation, Midland, MI, USA) was poured over a positive replica of cardiac tissue and polymerized for 4 h at 80 °C. After peeling off the PDMS-based negative replica from the PUA-based positive replica, the cardiac tissue-mimetic 3D replica was produced.

### 2.2. Fabrication of 3D Hydrogel Film Structure

To produce a hydrogel-based 3D tissue structure, we used alginate hydrogel as a model biopolymer. At first, alginate solution (1~3 wt%) was poured into a PDMS-based negative replica. Then, the sample was put into the oven (80 °C) overnight to dehydrate the alginate solution. After fully dehydrating the alginate solution, it was crosslinked using calcium chloride (CaCl_2_, 100 mM) for 30 min at room temperature. Since the alginate solution was immediately crosslinked by CaCl_2_, a thick hydrogel scaffold was fabricated rather than a thin hydrogel film as when CaCl_2_ was mixed in the dehydration process. In addition, although the CaCl_2_ had more difficulty in penetrating dehydrated alginate compared to alginate solution, 30 min were enough to crosslink dehydrated alginate. Finally, the cardiac structure-mimetic alginate film was fabricated.

### 2.3. Synthesis of Catechol-Conjugated Alginate (ALG-C)

ALG-C was synthesized via EDC-NHS chemistry. Briefly, 100 mg of alginate powder (Sigma-Aldrich, Oakville, ON, USA) was dissolved in 10 mL of distilled water. Then, 143 mg of 1-Ethyl-3-(3-dimethylaminopropyl) carbodiimide hydrochloride (EDC·HCl), 86 mg of N-hydroxysuccinimide (NHS), and 130 mg of dopamine (Sigma-Aldrich) were added to the alginate solution. The mixture was reacted overnight at room temperature. Then, unreacted EDC, NHS, and dopamine were dialyzed using dialysis membrane (MWCO: 12 K Da, Spectra/Por^®^) against distilled water for 3 days. The distilled water was changed three times each day. After lyophilization at −50 °C for 3 days (FreeZone 2.5 Liter Benchtop Freeze Dry System, Labconco), the alginate-catechol conjugate was collected. The degree of substitution was analyzed by ^1^H NMR spectroscopy (Avance III 400 FT-NMR, Bruker). Briefly, 5 mg of ALG-C was dissolved in 1 mL of deuterium oxide (D_2_O, Sigma-Aldrich). Then, ALG-C solution was loaded in Wilmad^®^ NMR tube and analyzed. In addition, the synthesis of ALG-C was further characterized by Attenuated Total Reflection-Fourier Transformation Infrared (ATR-FTIR, TENSOR27, Bruker). Briefly, the lyophilized ALG-C was scanned 32 times with a resolution of 8 cm^−1^ and measured from wavenumbers ranging from 650 to 4000 cm^−1^. The ALG-C film was also fabricated via the above process using CaCl_2_.

### 2.4. Scanning Electron Microscopy (SEM) Analysis

Lyophilized ALG and ALG-C films (1, 2, and 3 wt%) were mounted on aluminum stubs with carbon tapes. Then, platinum/palladium was coated on the surface of the sample using sputter for 110s in vacuo. The image was taken with SEM (JSM-7610F, JEOL, Tokyo, Japan) at 10 µA at 10 kV.

### 2.5. Measurement of Water Absorption

To measure the water absorption behavior of the hydrogels, each sample was fabricated as described above. Each sample was immersed in distilled water for 24 h at room temperature to reach equilibrium state. Then, the samples were lyophilized and weighed. The water absorption was calculated following this equation:

$$Water\;absorption=\frac{W_{s}-W_{d}}{W_{d}}$$

where *W_s_* and *W_d_* represent the weight of the swollen and dried states, respectively, at equilibrium condition.

### 2.6. Measurement of Calcium Content on Alginate Hydrogel and Alginate Film

To quantify calcium content on each sample, a fluorescence assay using Indo 1-AM (I3261, Sigma-Aldrich) was performed following the manufacturer’s protocol. Briefly, each hydrogel or film sample was smashed and incubated with Indo 1-AM solution (10 µM, dissolved in dimethyl sulfoxide (DMSO)) for 30 min at 37 °C. After being centrifuged for 5 min at 12,000 rpm, the supernatant was collected, and the fluorescence emission spectra were measured at 355 nm by Infinite 200 Pro microplate reader (Tecan). The unreacted calcium ions were detected at 460 nm.

### 2.7. Quantification of Young’s Modulus

Mechanical properties of the hydrogel films were evaluated using a Universal Tensile Machine (UTM, 100 N of load cell, EZ-SX STD, Shimadzu, Osaka, Japan). Briefly, the ALG and ALG-C films were prepared as mentioned above. The ALG hydrogels were immersed in phosphate buffer saline (PBS) to swell equilibrium. After removing residual PBS using Kimwipes, the sample was compressed with 3 mm/min of probe speed. As ALG-C film was too thin to do a compression test, we stretched ALG-C film with 3 mm/min of probe speed. To calculate Young’s modulus, we used a linear region of the stress–strain curve from 5% to 15%.

### 2.8. Determination of Degradation Kinetics

To determine the degradation kinetics of hydrogel film, each hydrogel film sample was immersed in PBS at 37 °C to swell equilibrium. After weighing the sample, each sample was treated with PBS at 37 °C. On days 1, 2, 5, and 7, samples were collected and measured at wet weight. The degradation of ALG and ALG-C film was calculated by the following equation:

$$\;Degradation\;rate=\frac{M_{t}}{M_{i}}$$

where *M_i_* and *M_t_* represented the wet weight at initial state and experimental time t, respectively.

### 2.9. Live/Dead Assay

To verify the biocompatibility of alginate film, we conducted a live/dead assay following the manufacturer’s protocol. Briefly, both C2C12 cells and H9c2 cells (cardiomyoblast, CRL-1446^TM^, ATCC) were cultured on alginate film (cell density: $1\times 10^{5}\;\mathrm{cells}/{\mathrm{cm}}^{2})$ in DMEM (HyClone, Logan, UT, USA) containing 10 wt% fetal bovine serum (FBS, Biowest, Nuaille, France), 1 wt% L-glutamine (Gibco, Grand Island, NY, USA), and 1 wt% penicillin-streptomycin (Gibco). Each cell was stained with 2 µM of ethidium homodimer-1 (EthD-1) and 4 µM of calcein-AM in a cell culture medium for 30 min. After washing three times with PBS for 5 min, images were taken by EVOS^®^ FL Cell Imaging System (Thermo Fisher, Cleveland, OH, USA). The cell viability was measured by calculating the number of calcein-stained cells (live cells) per total cell number using ImageJ (ImageJ software, version 1.53k14).

### 2.10. Synthesis of Lentiviral Vectors

Lentivirus was synthesized as previously described [1]. Briefly, lentiviral expression plasmid (PLV-eGFP, Addgene Plasmid 36083), packing plasmid (psPAX2, Addgene Plasmid 12260), and enveloping plasmid (pMD2.G, Addgene Plasmid 12259) were prepared in PBS and electroporated into HEK-293T under 1100 V, 2 ms, 2 pulses conditions. The supernatant cell medium was collected every 24 h for 3 days. After filtering with 0.45 µM cellulose acetate filter, the lentiviral vectors were concentrated by using PEG-it ^TM^ (System Biosciences, Mountain View, CA, USA) and centrifuged at 12,000 rpm for 30 min at 4 °C.

### 2.11. Determination of Drug Coating Efficiency

To determine drug coating efficiency on 3D tissue structure, we used dextran and lentivirus as model drugs. At first, fluorescein isothiocyanate-dextran (FITC-dextran, 1 wt%) and FITC-labeled lentivirus ($1\times 10^{9}\;\mathrm{particles}/\mathrm{mL}$) was coated on alginate and catechol-alginate film for 24 h at 4 °C, respectively. Then, unbound model drugs were vigorously rinsed three times with PBS for 5 min. The concentration of bound model drugs was calculated using fluorescent intensity at 475 nm by UV-spectroscopy (TECAN infinite m200 pro, Switzerland).

### 2.12. Statistical Analysis

All data were displayed as mean and standard deviation (SD). All trials were performed in triplicate. Statistical significance was calculated by one-way ANOVA and Student’s t-test with * *p* < 0.05, ** *p* < 0.01, or *** *p* < 0.001.

## 3. Results

### 3.1. Fabrication of 3D Hydrogel Film for Cardiac Tissue Engineering

**Scheme 1:**
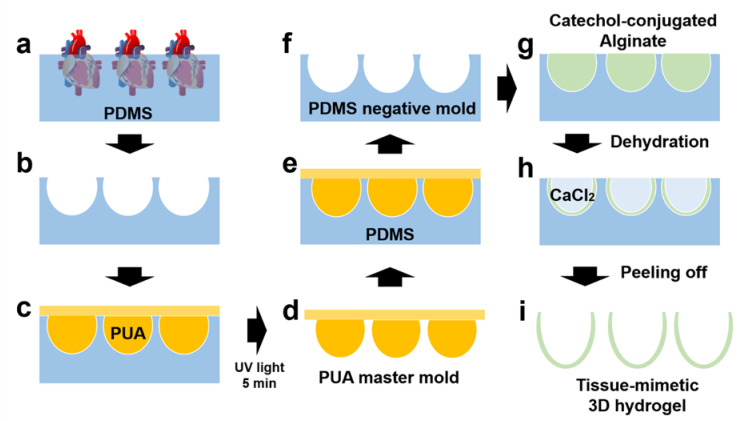
Schematic illustration of the fabrication procedure of 3D hydrogel film using the catechol-conjugated alginate (ALG-C) hydrogel.

**Figure 1:**
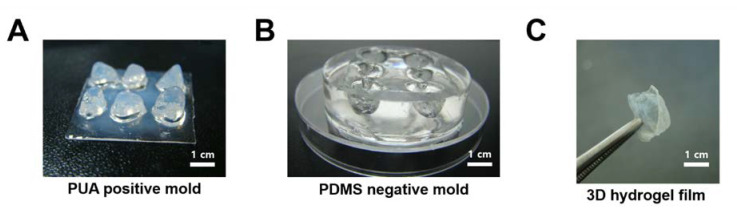
Photographic images of tissue-mimetic replica and 3D hydrogel structure. (**A**) Image of PUA-based cardiac-mimetic positive mold (replica). This positive replica was used as a master mold to produce PDMS-based negative mold. (**B**) Image of PDMS-based cardiac-mimetic negative mold. (**C**) Image of ALG-C film mimicking the structure of cardiac tissue surface.

In this study, we developed a 3D hydrogel film for drug delivery on cardiac tissue (Scheme 1). To fabricate 3D hydrogel film, we slightly modified the poly dimethyl siloxane (PDMS)-based conventional cast molding process [28,29]. First, we sacrificed balb-C mice (4 weeks old) to collect cardiac tissue. After being dried for 2 days at room temperature, it was put into a PDMS precursor, following curing PDMS for 4 h at 80 °C. After which, to make the cardiac shaped-master mold, we poured and cured polyurethane acrylate (PUA) (Figure 1A). Using a PUA-based master mold, we fabricated a PDMS-based negative replica to produce the hydrogel-based 3D film (Figure 1B). The catechol-conjugated alginate (ALG-C) was poured into a PDMS replica and dried overnight at 80 °C to get a dried ALG-C 3D film. A free-standing dried film was obtained without crosslinking, which was attributed to the concentration of polymeric solution; however, we further applied CaCl_2_ to fabricate crosslinked 3D film possessing mechanical stability (Figure 1C).

Recent advances in 3D printing have enabled the fabrication of 3D structures with soft materials such as hydrogels [30,31,32]. The 3D printing would also be able to reconstruct cardiac tissue with hydrogel when the structure of multi-scale cardiac tissue is recapitulated by non-invasive imaging tools such as computer tomography (CT) [33,34]. Several studies have demonstrated artificial whole heart chambers by seeding cardiomyocytes with hydrogel in a ventricle-like shaped mold or by using 3D printing technology [12,13,14,35,36,37]. Nevertheless, the technique to make a highly thin film with a multi-scale tissue structure has still been challenging. In addition, although substrate-mediated delivery of biological macromolecules into human tissue has been a prominent approach for tissue engineering, a method for fabricating complex-shaped hydrogel substrate in 3D has not been well established. Remarkably, our strategy of dehydration of hydrogel precursor on the surface of a complex structure is a novel methodology to make a 3D hydrogel structure efficiently in a short time with low cost. The preparation of dry alginate film has already been introduced in membrane-based separation processes and food packaging [38,39,40]. Previous studies demonstrated that alginate film fabricated by evaporation has a thickness of less than 50 µm and high water permeability, suitable for biomedical applications [38,39,40]. By combining both simple dehydration of hydrogel precursor and 3D tissue replica, we could easily fabricate hydrogel film in 3D that recapitulated surface topography of native tissue. This methodology can be applied to fabricate other types of tissue with different hydrogel precursors.

### 3.2. Physicochemical Properties of the 3D Hydrogel Film

**Figure 2:**
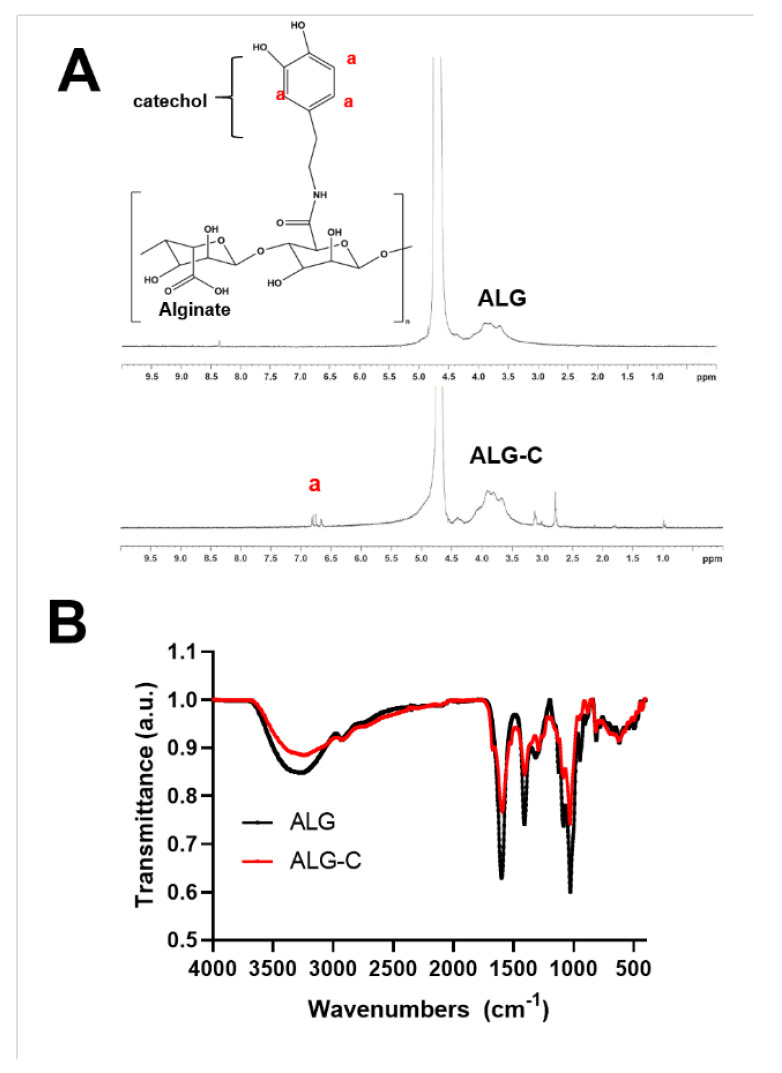
Detection of catechol moiety on ALG-C by ^1^H NMR and ATR-FTIR. (**A**) The chemical shift of two H at the ortho-position and meta-position represented around 6.5 ppm and 6.9 ppm, respectively. (**B**) The ATR-FTIR spectra showed the successful conjugation of catechol onto the alginate backbone.

Next, we analyzed the physicochemical properties of the 3D hydrogel film. In this study, we used ALG-C hydrogel as a drug delivery carrier. Hydrogel functionalization with catechol groups has several advantages: it has (i) biocompatibility, (ii) non-specific interaction with biological macromolecules and (iii) tissue adhesiveness [41,42]. In terms of drug delivery, the carrier should capture the drug molecules efficiently and release them properly when delivered [43,44]. As the catechol group can interact with biological molecules via non-covalent bonds, a catechol-conjugated hydrogel film can load drug molecules on the surface of the substrate without any additional chemical reaction or treatment. To analyze physicochemical properties of the 3D hydrogel film, we confirmed the conjugation of the catechol group on the ALG (Figure 2). The catechol group was easily functionalized on carboxylic acid of alginate via EDC/NHS chemistry. When we measured the chemical shift using nuclear magnetic resonance (NMR), ALG-C exhibited peaks at 6.5–6.8 ppm, indicating the catechol protons. Furthermore, Figure 2B shows the FT-IR spectra of both ALG and ALG-C. The ALG represented a signal at 3200–3600 cm^−1^ attributed to the -OH stretching vibration. The peaks at 1610 and 1421 cm^−1^ showed asymmetric and symmetric stretching of carboxylic salt ion. The peak at 1035 cm^−1^ corresponded to the C-O stretching of the pyranose ring. In the ALG-C spectrum, the broad peak at -OH stretching declined, while the peak of the phenolic structure of the catechol groups at 1279 cm^−1^ and 780 cm^−1^ appeared. Through the analysis of 1H NMR and FT-IR spectrum, we could finally prove that the catechol groups were successfully conjugated onto the alginate.

**Figure 3:**
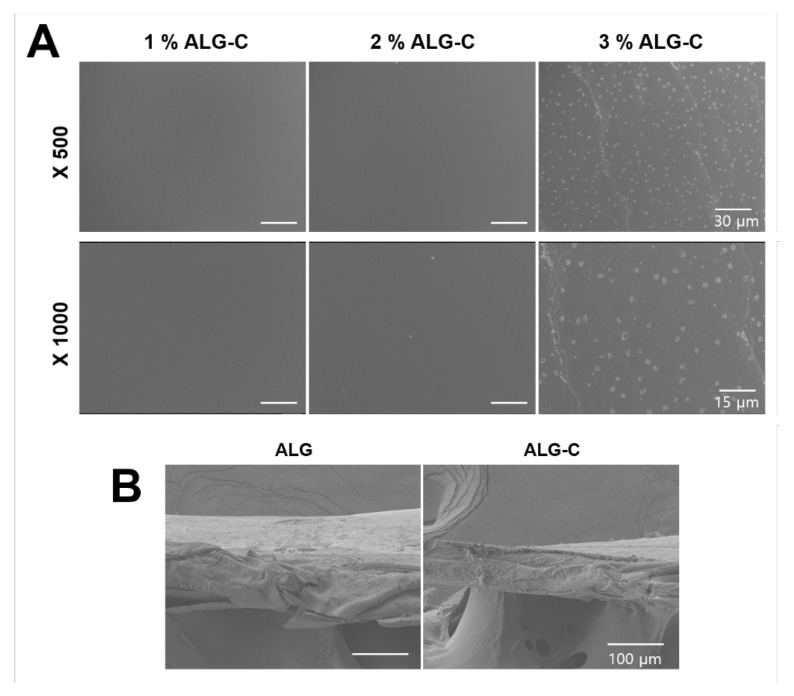
SEM images of films with 3 levels of ALG-C concentrations. (**A**) The ALG-C film with 1% and 2% had a flat and flawless surface, but 3% ALG-C showed a wrinkled surface and lots of aggregates. (**B**) The cross-sectional image of both ALG and ALG-C shows the films had a thickness lower than 100 μm.

Next, we imaged the surface of the hydrogel substrate in a microscopic view. Interestingly, 3.0% of ALG-C had numerous aggregates over the surface, which was not observed in the 1.0% and 2.0% ALG-C groups (Figure 3A). Since these aggregates might affect the loading efficacy of drugs, we selected the 2.0% ALG-C film that had a flawless surface and conducted the following experiments. In addition, we identified the film thickness by observing the cross-section plane. The thickness of both ALG and ALG-C film was lower than 100 μm (Figure 3B). Considering that the diffusion limitation of oxygen and nutrients is around 200 μm [45], ALG-C satisfied the metabolic demand of cells.

**Figure 4:**
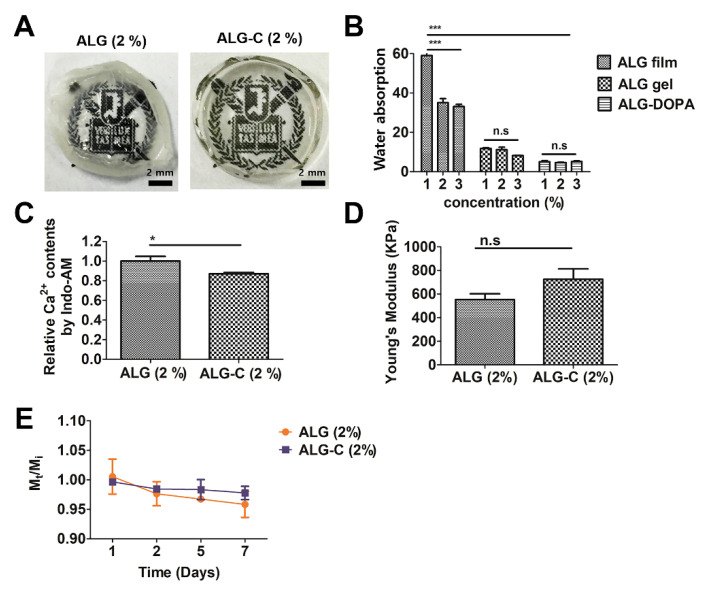
Optical property and water absorption of both ALG and ALG-C films. (**A**) Photographic image of the 3D-structured films. The ALG-C has higher optical transparency than ALG. (**B**) The water absorption of conventional ALG hydrogel, ALG films, and ALG-C films. (**C**) Relative Ca^2+^ ion concentration within the films was measured by Indo-AM assay. (**D**) Quantification of Young’s modulus of ALG and ALG-C films. (**E**) Degradation kinetics of ALG and ALG-C films. All the experiments were conducted in triplicate (* *p* < 0.05, *** *p* < 0.001).

Next, we analyzed the crosslinking properties of hydrogel substrate with ALG-C (Figure 4). Compared to ALG film, the ALG-C film has higher optical transparency, which allows the background image to be observed through the film (Figure 4A). ALG-C film also has a rigid structure that can be free-standing. Generally, the hydrogel can absorb large amounts of water. The alginate hydrogel crosslinked by calcium ions exhibited water absorption from 35 to 60 depending on alginate concentrations (Figure 4B); however, the water absorption property of ALG film was dramatically changed. The ALG film, fabricated by dehydration, could absorb water about 6 times less than conventional ALG hydrogel. Especially, the ALG-C film had 12 times lower water absorption. We speculated that as the alginate was highly concentrated after being dehydrated, the water-absorbing capacity inside the polymer chain was significantly decreased. Furthermore, the calcium content in the ALG-C film were lower than that of the ALG film (Figure 4C). The catechol group in hydrogel can be oxidized by residual oxygen to form a reactive quinone adduct [20,46]. The quinone group immediately coupled with other phenolic moieties, resulting in crosslinking hydrogel. Although the lower content of calcium ions can imply a decrease in crosslinking density, the automatic oxidation of the catechol group in ALG-C film acts as a secondary crosslinker, suggesting that the ALG-C film has a dual crosslinking system that consists of ionic and oxidative crosslinking. For this reason, although the ALG-C group has lower calcium content than the ALG group, it has a more rigid structure in terms of mechanical properties. We also confirmed the mechanical properties of ALG and ALC-C films (Figure 4D, Supplementary Figure S1). In previous studies, polymer-based scaffolds around the MPa scale can support cardiomyocytes growth in vitro and in vivo [13,47,48]. Young’s modulus of ALG and ALG-C film was 552.8 ± 70.15 kPa and 725.2 ± 123.4 kPa, respectively.

Next, we determined the degradation kinetics of ALG and ALG-C (Figure 4E). All the samples maintained inter-network structure until 1 week in PBS at 37 °C without significant differences between groups. The alginate hydrogel is usually degraded by ionic diffusion from the hydrogel network to the surrounding medium and fully removed in months in vivo [49]. To facilitate kinetics of degradation, matrix metalloproteinase (MMP) with catechol di-terminated can be utilized in our ALG-C film [20,50,51]. We expected that catechol di-terminated MMP peptides might regulate the biodegradability of ALG-C film in terms of proteolytic degradation.

### 3.3. Determination of Drug Loading Efficacy

**Figure 5:**
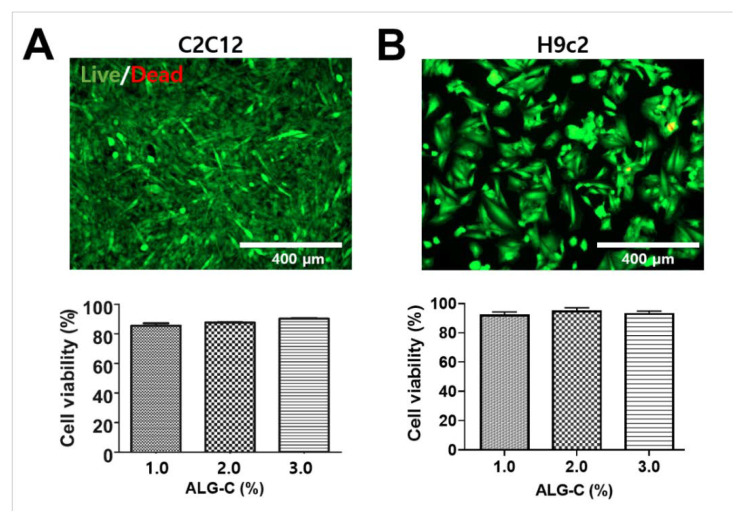
Biocompatibility of ALG-C film hydrogel. Fluorescence image of live (green) and dead (red) cells on the 2% ALG-C film using (**A**) C2C12 cells and (**B**) H9c2 cells. The cell viability was quantified according to ALG-C concentration. All the experiments were conducted in triplicate.

When the substrate is delivered to the target tissue, endogenous cells migrate from native tissue to the substrate and directly interact with drug molecules [52,53]. Therefore, we confirmed the biocompatibility of the films with a Live/Dead assay using both C2C12 cells and H9c2 cells. In cardiac tissue, there are three representative cell types: (i) cardiomyocytes, (ii) endothelial cells, and (iii) stromal cells [54,55]. The use of cardiomyocytes for cell transplantation and modeling in vitro cardiac tissues has been widely studied and recognized as a promising approach [56,57,58]. The cardiomyoblast H9c2 cell line, which can be differentiated into functional cardiomyocytes, has been proved in several studies as a candidate cell source for cardiac tissue engineering [25,26,27]. All the groups showed high cell viability over 90% (Figure 5).

**Figure 6:**
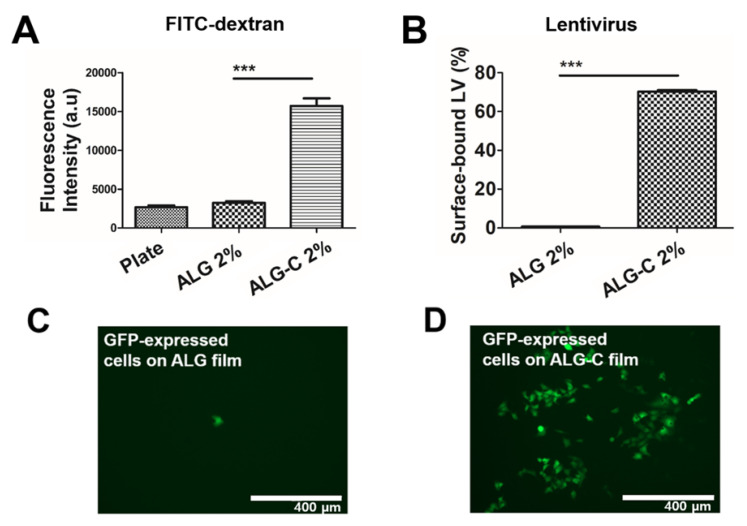
The drug loading efficacy of ALG-C film. The ALG-C film showed a significantly higher drug loading efficacy of both (**A**) FTIC-dextran and (**B**) lentivirus than that of ALG film. Representative images of the transfected cells on the lentivirus-coated (**C**) ALG film and (**D**) ALG-C film. All the experiments were conducted in triplicate. (*** *p* < 0.001).

Next, we evaluated drug loading efficacy on the substrate using two different types of model drugs, i.e., FITC-dextran and lentivirus. FITC-dextran is a representative model of peptide drugs that have been widely used for drug release tests [59]. The quantification of fluorescence intensity of surface-bound FITC-dextran exhibited that ALG-C film could easily capture the FITC-dextran compared to ALG film (Figure 6A). However, the ALG film and control group (plate) showed a similar degree of fluorescence intensity, indicating non-specific binding of FITC-dextran. In addition, the ALG film only captured less than 1% of lentiviral particles, but the ALG-C film has around 70% of lentiviral particles on the surface after harsh washing with PBS (Figure 6B). This is because the catechol groups can interact with the outer layer of lentivirus, mostly comprised of capsid proteins, through non-specific interaction [60,61]. Subsequently, GFP gene-encoded lentiviral particles on ALG-C film efficiently transfected into the C2C12 cell line expressing green fluorescence (Figure 6C,D). It demonstrated that those catechol residues of ALG-C effectively interacted with FITC-dextran and lentivirus acting as a good carrier of biological macromolecules.

## 4. Conclusions

In this study, it was demonstrated that a simple direct tissue casting method reconstructed a complex tissue morphology with hydrogel in 3D for drug delivery. In making full use of the PDMS, the surface shape of complex cardiac tissue could be easily reproduced. By combining tissue mold and catechol-functionalized alginate, catechol-derivatives can also provide cell-friendly environments supporting biological cues on alginate hydrogel. Since biocompatible alginate film does not provide appropriate biological signals for cellular activity, the presentation of the catechol group on alginate has enhanced cellular attachment and ECM deposition. Along with the high cell viability of H9c2, ALG-C film can capture a high degree of model drugs such as FITC-dextran and lentivirus through catechol moieties. Our work suggests that functionalized hydrogel film in 3D with a complex tissue morphology has a robust potential for cardiac tissue engineering as well as macromolecule delivery.

## Supplementary Materials

The following are available online at https://www.mdpi.com/article/10.3390/bioengineering8110164/s1, Figure S1: Tensile test of ALG-C film.
