# Supplementary material for: Facile Fabrication of Three-Dimensional Hydrogel Film with Complex Tissue Morphology
Source: Bioengineering (Basel). 2021 Oct 27;8(11):164. doi: 10.3390/bioengineering8110164 (PMC8614799; doi:10.3390/bioengineering8110164)
Supplement: Supplementary file 1 [file bioengineering-08-00164-s001.zip › bioengineering-1387319-supplementary.pdf]

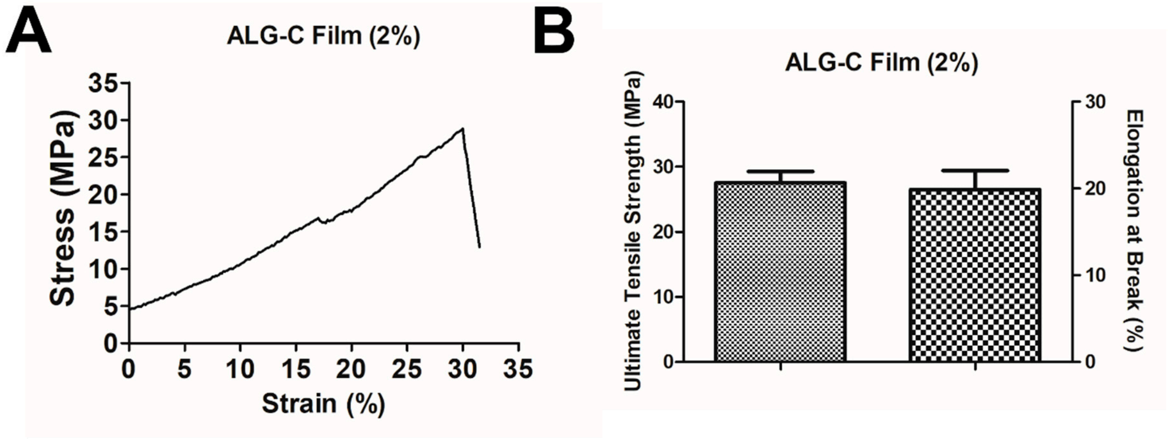

Supplementary Figure S1. Tensile test of ALG-C film. (A) Stress-strain curve of ALG-C film. (B) Evaluation of ultimate tensile strength and elongation at break. All trials were performed in triplicate.
